# Ketogenic Diet Versus Immunological Therapy in the Management of Refractory Epilepsy in Children

**DOI:** 10.7759/cureus.103310

**Published:** 2026-02-09

**Authors:** Osama M Elasheer, Emad H Eldaly, Eman F Gad, Nancy A Elgalaly, Duaa M Raafat, Noha ElGyar

**Affiliations:** 1 Pediatrics, Assiut University, Asyut, EGY; 2 Pediatric Neurology, Assiut University, Asyut, EGY; 3 Pediatric Medicine, Assiut University, Asyut, EGY

**Keywords:** children, epilepsy, immunological, ketogenic diet, refractory, rituximab

## Abstract

Background: In neuroclinical practice, refractory epilepsy can be identified only after the failure of several antiepileptic drugs (AEDs), so newer lines of treatment have been used, such as the ketogenic diet and rituximab.

Methods: A total of 105 patients with refractory epilepsy were divided into three equal groups, each receiving a specific treatment: group R received rituximab, group K received a ketogenic diet, and group S received steroids for six months. Seizure frequency was recorded before and after enrollment.

Results: The age to start AEDs, the age at enrollment in the study, and the duration of use of AEDs were comparable among the three groups. Family history was the highest in rituximab and consanguinity was the highest in steroid but without significant statistical difference with p value 0.829 and 0.941 respectively. There was a significant improvement (in the form of fit reduction) in all groups with a p-value of <0.001. This percentage of change was higher in rituximab (42.9%), followed by ketogenic diet (30%), then steroid (21%). The presence of a positive family history of epilepsy had a significant effect on improvement using any line of treatment, with a p-value of 0.022. Patients with a positive family history of epilepsy or positive consanguinity or multiple types of convulsions showed significant improvements with ketogenic drugs compared to other patients, with p-values of 0.012, 0.044, and <0.001, respectively.

Conclusion: Adding rituximab or a ketogenic diet to patients with refractory epilepsy provides better control of fits, with percentage improvements of 42.9% and 30%, respectively.

## Introduction

In general, definitions of refractory epilepsy consider the number of drug failures, the resulting frequency of convulsions per day, and the time required to achieve this outcome [1]. In 2011, the International League Against Epilepsy (ILAE) proposed that the presence of seizures within a six-month time frame, despite the use of appropriate therapeutic regimens (either monotherapy or combination), should be standardized as the definition of pharmacoresistant epilepsy [2]. Recently, up to 20% of children have pharmacoresistant epilepsy, which reaches up to 40% in adult epileptic patients [1]. Brodie et al. analyzed the response of epileptic patients to anticonvulsant medications (ASMs) and found that 49.5% and 36.0%, respectively, responded favorably to the first and second drug regimens. Consequently, subsequent to the ineffectiveness of the initial two pharmaceuticals, all therapies incorporating ASM exhibited a diminished rate of success [3]. According to the comorbidities of the patients, IV methylprednisolone (IVMP) or intravenous immunoglobulin (IVIg) can be used as first-line therapy for refractory epilepsy patients [4].

The pathogenesis and clinical manifestations of autoimmune epilepsy, which responds favorably to immunosuppressive therapies [5], have been the subject of increased investigation. Certain patients with AE do not exhibit a positive response to immunotherapy; rather, they may require costly, intensive, long-term treatment. For this reason, scholars are investigating approaches to promptly diagnose adverse events (AEs) and develop predictive models to identify autoantibodies based on neurological assessments and presentation prior to testing [6].

A model known as the Antibody Prevalence in Epilepsy and Encephalopathy (APE2) score was recently devised to forecast the occurrence of autoimmune epilepsy by utilizing clinical attributes. Refractory epilepsy patients are thought to benefit therapeutically from the ketogenic diet (KD), modified Atkins, and low glycemic index diets. Most patients on the modified Atkins diet show a six-month improvement in seizure frequency of more than 50%. Additionally, behavioral and developmental changes were noted. The benefits of the KD can remain even after one returns to a regular diet [7]. A ratio of calories from fat to non-fat represents the KD prescription. Starting in a 1:1 ratio, it is progressively increased to 3:1 or 4:1 based on urine ketosis, seizure reduction, and palatability. The mechanisms of action of the KD as an anti-epileptogenesis include ketosis, enhanced mitochondrial biogenesis and oxidative phosphorylation, increased gamma-aminobutyric acid (GABA) levels, and decreased neural excitability and firing [8].

Many inflammatory and autoimmune disorders have been treated initially with corticosteroids. For autoimmune epilepsy, the suggested dosage is 30 mg/kg/day, up to 1,000 mg/day IV infusion given every three to five days. An empirical steroid may worsen a condition such as an infection. Steroids also cannot work well against immunological disorders caused by antibodies. Corticosteroid adverse effects, such as delirium, depression, anxiety, and sleep loss, can also influence the treatment response. Rituximab, a monoclonal antibody, binds to CD 20 on the surface of B cells. Although rituximab has been used as a second-line treatment, its administration in combination with steroids or IVIg has been investigated because of its positive results in autoimmune encephalitis. Rituximab lowers the recurrence rate and speeds up healing. Although a 375 mg/m^2^ weekly IV infusion for four weeks is the usual advised regimen, the patient's condition can dictate the dose and interval (750 mg/m^2^ every two weeks, twice) [9]. More randomized studies are necessary because it is currently unknown if a monthly IVIg-boosting infusion and the monthly maintenance of rituximab are advantageous for recovery.

The main objective of the research was to assess and compare the effectiveness of rituximab, steroids, and a KD in managing refractory epilepsy. Recent reports suggest that in patients with refractory epilepsy who are taking well-tolerated, appropriate anticonvulsants, the addition of rituximab results in improved seizure control with minimal adverse effects.

## Materials and methods

Study design and setting

The current study is a randomized clinical trial. The study was done at the Pediatric Neurology Unit in Assuit University Children Hospital (AUCH), Asyut, Egypt, in the period from October 2020 to October 2021. The study protocol was approved by the Ethical Committee in the Faculty of Medicine at Assiut University with the approval number 17200503. The authors registered the trial at ClinicalTrials.gov with ID number NCT (04542629) and followed the Consolidated Standards of Reporting Trials (CONSORT) statement. Informed written consents were obtained from the parents of patients to conduct this research before enrollment.

Patient selection

The inclusion criteria were patients aged one to 17 years, both males and females, with epilepsy who were receiving two tolerated and appropriately chosen antiepileptic drugs (AEDs) without response for a duration of six months, according to the 2009 ILAE guidelines, and including all types of epilepsy.

Exclusion criteria

Participants were excluded if they had an inappropriate diagnosis mimicking epilepsy or leading to pseudo-refractoriness, such as cardiogenic events, vasovagal syncope, parasomnias, movement disorders, or psychogenic non-epileptic seizures. Poor medication compliance with missed doses was also grounds for exclusion. Participants prescribed an incorrect AED, receiving inadequate AED dosage or frequency, or experiencing enzyme induction, especially with multiple AEDs or medications, were excluded. These criteria aimed to represent the target population accurately, without confounding factors like misdiagnosis, non-compliance, or suboptimal treatment regimens.

Methods

The evaluation of patients with epilepsy involved a comprehensive approach, starting with a detailed history of the onset, description of seizures (pre-ictal, ictal, and post-ictal events), precipitating factors, history of status epilepticus (SE), relationship to fever or previous febrile seizures, history of brain insults (trauma, intracranial hemorrhage, encephalitis), family history (consanguinity, similar conditions, affected siblings), developmental history, sleep history, and detailed birth and perinatal history. Previous AED use was also documented. Clinical examination focused on identifying specific facial features or skin lesions suggestive of neurocutaneous syndromes, along with a detailed neurological and systemic assessment. Investigations included electroencephalogram (EEG), renal and liver function tests, serum electrolytes and glucose levels, computed tomography (CT) or magnetic resonance imaging (MRI) of the brain, metabolic workup (arterial blood gas, serum ammonia, lactate, tandem mass screening), genetic testing (karyotyping, gene sequencing), fundus examination if needed, and cerebrospinal fluid (CSF) culture and analysis if indicated.

The practice recommendations for AED trials involved optimizing the dose of each AED by incremental increases. If the maximum dose was ineffective, a second AED was added while continuing the first one. If seizure control was achieved, tapering of the first AED was considered. If one or two AEDs were ineffective, rational polytherapy with different mechanisms of action was explored, avoiding AEDs that could worsen or provoke seizures. The treatment plan involved dividing patients into three groups, each receiving rituximab, corticosteroids, or a KD. The choice of treatment line was randomized, considering drug availability, contraindications, and patient preference. Corticosteroid therapy involved a pulse dose of 30 mg/kg/day for five days (maximum 1 g/day), given monthly for six months.

For rituximab, patients were admitted to the hospital for consent and fitness evaluation, including a tuberculin test, complete blood count, renal and liver function tests, electrolytes, hepatitis B and C screening, electrocardiogram, and chest X-ray. Contraindications included hypersensitivity to rituximab, heart failure, active infection, and hepatitis B. Pre-medication with methylprednisolone, chlorphenamine, and paracetamol was given before each rituximab infusion. The initial dose was 750 mg/m^2^ every two weeks for two doses, followed by maintenance doses of 750 mg/m^2^ every six months.

For the KD, patients were admitted for fitness evaluation, including complete blood count, fasting blood sugar, electrolytes, liver and kidney function tests, lipid profile, urinalysis, and tandem mass screening or organic acid analysis if needed. Contraindications included carnitine deficiency, carnitine palmitoyl transferase I or II deficiency, carnitine translocase deficiency, β-oxidation defects, pyruvate carboxylase deficiency, and porphyria. Precautions included converting all medications to tablet form before starting the diet. Caloric and protein requirements were calculated, and patients were started on a classic KD with a 3:1 or 4:1 ratio (grams of fat to combined carbohydrate and protein), gradually increasing the fat content over three days. Special milk formula was provided for children under two years, while older children received a low-carbohydrate diet planned according to socioeconomic status, food availability, and maternal education. Follow-up included monitoring for acidosis, ketone levels, dietary compliance, and regular visits. After six months, patients were evaluated for seizure frequency, AED types, and doses.

Sample size

G*Power 3 software 3.1.9.2 (Heinrich-Heine-Universität, Düsseldorf, Düsseldorf, Germany) was used to calculate the sample size. To find an effect size of 0.5, a minimum calculated sample of 105 patients is needed with an error probability of 5% and 95% power. The main statistical test is the t-test to detect the differences between the two groups. The significance level (α) was set at 0.05, the statistical power at 0.80, the effect size at 0.7, and the allocation ratio at 1:1. A total of 105 patients with refractory epilepsy (35 in each group) were included in our study.

Statistical analysis

IBM SPSS Statistics for Windows, Version 22 (Released 2013; IBM Corp., Armonk, New York, United States) was used for statistical calculations. Non-normally distributed data are presented as mean ± SD, median, range, frequencies, and relative frequencies (percentages) when applicable. Kruskal-Wallis and Mann-Whitney U tests were used to compare quantitative variables since the data were not regularly distributed. The Wilcoxon signed-rank test compared paired quantitative data. To compare categorical data, the chi-square (χ^2^) test was used. For anticipated frequencies less than 5, an exact test was employed. The p-value is always two-tailed and significant at 0.05.

## Results

A total of 105 patients with refractory epilepsy, aged one to 11 years, were enrolled in this randomized, single-blind study. Patients were allocated into three groups (35 per group): KD (K), rituximab (R), and steroid (S).

Table 1 shows that demographic data, risk factors, and clinical presentation were comparable among the three groups. The use of three or more AEDs did not differ significantly between groups (p = 0.588).

**Table 1:** Demographic data of the patients with epilepsy Statistical test: Kruskal-Wallis test. Statistical significance was set at p ≤ 0.05.

Variables				Groups			p-value
				Keto (N=35)	Rituximab (N=35)	Steroid (N=35)	
Demographic data							
Age (years) at enrollment to study				6.0 (1.0-11.0)	6.0 (1.8-17.0)	6.5 (2.0-15.0)	0.873
Age at start of treatment (years)				1.0 (at birth-6.0)	1.2 (1 month-14.0)	1.2 (1 month-14.0)	0.385
Duration of use of antiepileptic drugs (years)				4.0 (at birth-10.4)	2.8 (at birth-14.5)	2.3 (at birth-10.5)	0.372
Gender			Male	25 (71.4%)	20 (57.1%)	24 (68.6%)	0.658
			Female	10 (28.6%)	15 (42.9%)	11 (31.4%)	
Presence of a family history of epilepsy				14 (40.0)	19 (54.3%)	12 (34.3%)	0.829
Presence of consanguinity status				24 (68.5%)	22 (62.9%)	25 (71.4%)	0.941
Risk factors							
Status epilepticus				26 (74.3%)	28 (80.0%)	24 (68.5%)	0.549
Multiple types of convulsions				29 (82.9%)	29 (82.9%)	26 (74.3%)	0.585
With fever				24 (68.6%)	19 (54.3%)	22 (62.9%)	0.464
Convulsion during sleep				0 (0.0%)	2 (5.7%)	1 (2.9%)	0.357
Significant perinatal history	Normal			34 (97.1%)	33 (94.3%)	35 (100.0%)	0.601
	Preterm			0 (0.0%)	2 (5.7%)	0 (0.0%)	
	Neonatal convulsion			1 (2.9%)	0 (0.0%)	0 (0.0%)	
History suggesting a brain insult				0 (0.0%)	0 (0.0%)	0 (0.0%)	-
Clinical findings							
Hydrocephalus				1 (2.9%)	1 (2.9%)	0 (0.0%)	0.711
Tuberous sclerosis				1 (2.9%)	2 (5.7%)	1 (2.9%)	0.600
Hemi atrophy				0 (0.0%)	1 (2.9%)	1 (2.9%)	0.811
Neurofibromatosis				2 (5.7%)	1 (2.9%)	2 (5.7%)	0.792
Dysmorphic facies (microcephaly, depressed nasal bridge, cleft lip and palate, microphthalmia, hypertelorism)				6 (17.1%)	4 (11.4%)	5 (14.3%)	-
Hemiparesis (Rasmussen encephalitis)				1 (2.9%)	0 (0.0%)	1 (2.9%)	0.601
Medications							
Anti-epileptic medications		≤3 drugs		24 (68.6%)	22 (62.9%)	26 (74.3%)	0.588
		>3 drugs		11 (31.4%)	13 (37.1%)	9 (25.7%)	

Table 2 presents the developmental status of the patients, categorized as normal or abnormal, which was comparable among the three groups. Generalized tonic-clonic seizures were more frequent than focal and myoclonic seizure types, while absence seizures were observed in a small number of patients. Many patients exhibited more than one seizure type, resulting in overlap among seizure classifications.

**Table 2:** Neurological assessment among the studied groups Data are presented as median (interquartile range) or number (%). Statistical test: Kruskal-Wallis test. Statistical significance was set at p ≤ 0.05.

Variables	Groups			p-value
	Keto (N=35)	Rituximab (N=35)	Steroid (N=35)	
Developmental problems				
Normal	23 (65.7%)	18 (51.4%)	16 (45.7%)	0.223
Abnormal	12(34.3%)	17 (48.6%)	19 (54.3%)	
Delay	5 (14.3%)	7 (20.0%)	13 (37.1%)	-
Regression	7 (20.0%)	10 (28.6%)	6 (17.1%)	
Types of convulsion				
Generalized	23 (65.7%)	30 (85.7%)	22 (62.9%)	0.065
Focal	22 (62.9%)	24 (68.6%)	15 (42.9%)	0.072
Myoclonic	7 (20.0%)	6 (17.1%)	7 (20.0%)	0.942
Absence	0 (0.0%)	3 (8.6%)	0 (0.0%)	0.054

Table 3 presents the complete workup performed for the patients. Metabolic screening, ammonia level, lactate, and glucose were comparable among the three studied groups. A fatty acid oxidation defect was detected in 14% of patients who received rituximab. Brain CT, MRI, and EEG were performed for all patients, and there was no significant difference between normal and abnormal findings among the three groups. Abnormal CT brain findings were in the form of atrophy (20 patients), followed by Dandy-Walker malformation, hydrocephalus, and tuberous sclerosis (two patients each). MRI brain abnormalities included atrophy (22 patients), mesial temporal sclerosis (seven patients), followed by encephalitis (four patients). Abnormal EEG findings were in the form of multifocal discharges (24 patients), encephalopathy (19 patients), and centrotemporal spikes (13 patients).

**Table 3:** Laboratory and imaging data among the studied groups Note: Liver function tests, renal function tests, lipid profile, and arterial blood gas analysis were normal in all patients. ^#^ Metabolic testing was not performed in 42 cases. Data are presented as median (interquartile range) or number (%). Statistical test: Kruskal-Wallis test. Statistical significance was set at p ≤ 0.05.

Variables		Groups			p-value
		Keto (N=35)	Rituximab (N=35)	Steroid (N=35)	
Laboratory data					
Glucose		80.0 (60.0-140.0)	88.0 (40.0-100.0)	80.0 (60.0-100.0)	0.873
Ammonia		66.0 (45.0-100.0)	65.0 (50.0-100.0)	70.0 (40.0-90.0)	0.736
Lactate		13.0 (7.0-30.0)	12.0 (10.0-16.0)	12.0 (7.0-18.0)	0.783
Metabolic^#^	Normal	21 (100.0%)	18 (85.8%)	21 (100.0%)	0.054
	Fatty acid oxidation defect	0 (0.0%)	3 (14.2%)	0 (0.0%)	
	Organic academia	0 (0.0%)	0 (0.0%)	0 (0.0%)	
Imaging					
CT brain					0.711
Normal		26 (74.3%)	24 (68.5%)	27 (77.1%)	
Abnormal finding		9 (25.7%)	11 (31.5%)	8 (22.8%)	
Atrophy		8 (22.9%)	8 (22.9%)	4 (11.4%)	
Dandy-Walker		0 (0.0%)	2 (5.7%)	0 (0.0%)	
Hypoxic ischemic		1 (2.9%)	0 (0.0%)	0 (0.0%)	
Encephalomalacia		0 (0.0%)	1 (2.9%)	0 (0.0%)	
Tuberous sclerosis		0 (0.0%)	0 (0.0%)	2 (5.7%)	
Hydrocephalus		0 (0.0%)	0 (0.0%)	2 (5.7%)	
MRI					0.125
Normal		24 (68.2%)	17 (48.6%)	21 (60.0%)	
Abnormal finding		11(31.4%)	18 (51.4%)	14 (40.0%)	
Atrophy		8(22.9%)	8 (22.9%)	6 (17.1%)	
Dandy-Walker		0 (0.0%)	2 (5.7%)	0 (0.0%)	
Hypoxic ischemic		1 (2.9%)	0 (0.0%)	0 (0.0%)	
Encephalomalacia		0 (0.0%)	1 (2.9%)	0 (0.0%)	
Demyelination		2 (5.7%)	0 (0.0%)	0 (0.0%)	
Mesial temporal		0 (0.0%)	3 (8.6%)	4 (11.4%)	
Encephalitis		0 (0.0%)	3 (8.6%)	0 (0.0%)	
Limbic encephalitis		0 (0.0%)	1 (2.9%)	0 (0.0%)	
Tuberous sclerosis		0 (0.0%)	0 (0.0%)	2 (5.7%)	
Hydrocephalus		0 (0.0%)	0 (0.0%)	2 (5.7%)	
Rasmussen encephalitis		0 (0.0%)	0 (0.0%)	0 (0.0%)	
Polymicrogyria		0 (0.0%)	0 (0.0%)	0 (0.0%)	
EEG					0.861
Normal		9 (25.7%)	8 (22.9%)	10 (28.6%)	
Abnormal findings		26 (74.3%)	27 (77.1%)	25 (71.4%)	
Multifocal		8 (22.9%)	9 (25.7%)	7 (20.0%)	
Encephalopathy		6 (17.1%)	6 (17.1%)	7 (20.0%)	
Electrical status		4 (11.4%)	2 (5.7%)	3 (8.6%)	
Hypsarrythmia		2 (5.7%)	3 (8.6%)	3 (8.6%)	
Focal discharge		2 (5.7%)	2 (5.7%)	4 (11.4%)	
Centrotemporal spikes		4 (11.4%)	5 (14.2%)	4 (11.4%)	

**Figure 1:**
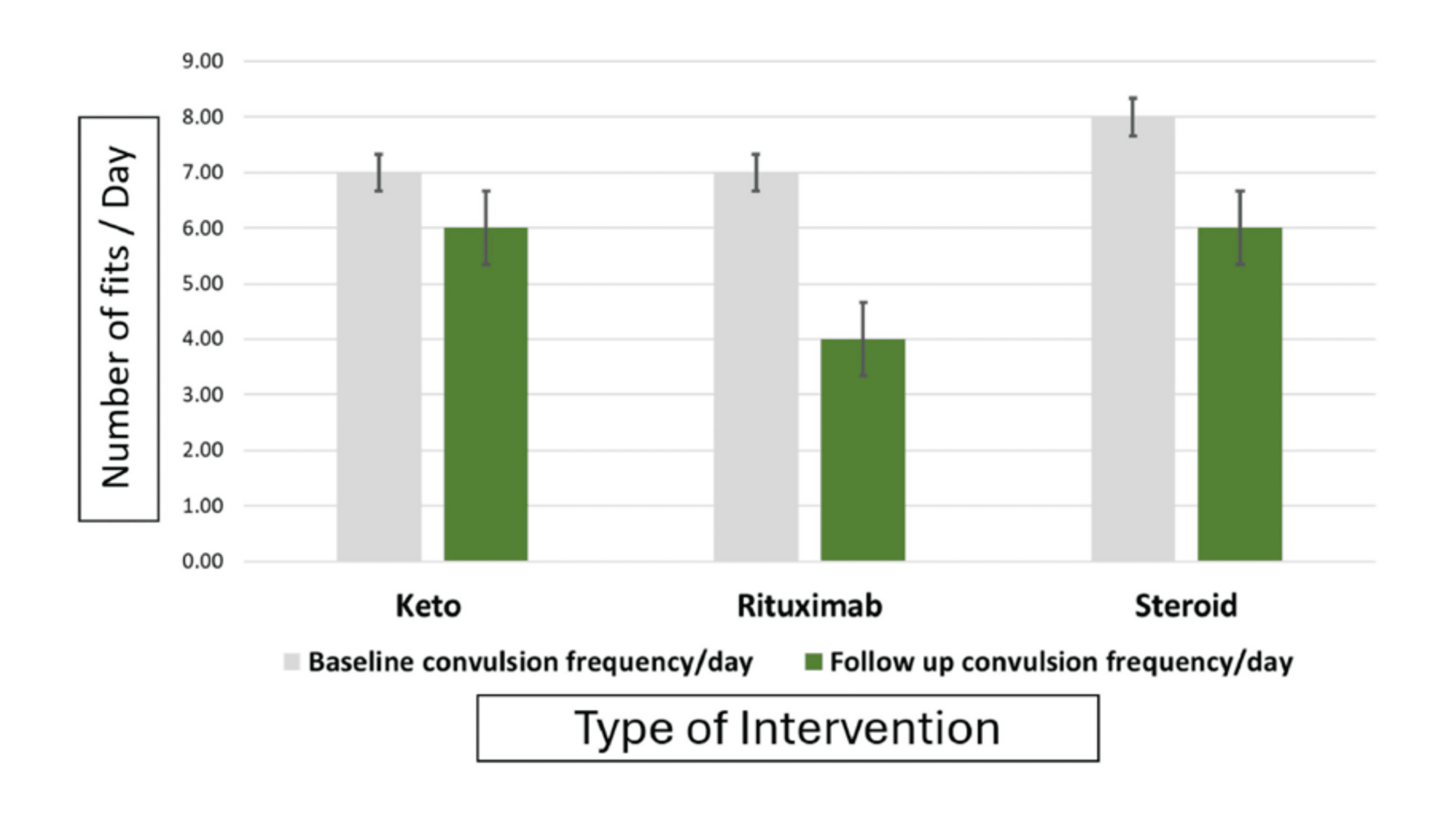
Frequency of convulsions at baseline and after treatment among the studied participants according to the treatment protocol

Figure 1 shows the frequency of convulsions from baseline to after treatment among the studied participants according to the treatment protocol. At baseline, the median convulsion frequency per day was similar between groups, with a median of 7 convulsions per day (interquartile range (IQR) 6-10) in the KD and rituximab groups and 8 convulsions per day (IQR 6-10) in the steroid group (p = 0.342). The figure also shows that after treatment, the median convulsion frequency was significantly reduced in all groups (p < 0.001 within each group). The rituximab group showed the greatest decrease, reaching 4 convulsions per day (IQR 3-6), compared to 6 per day (IQR 4-7) in both the KD and steroid groups. This difference between groups was statistically significant (p < 0.001). In terms of percent change from baseline, convulsion frequency decreased by 42.9% with rituximab, 30.0% with the KD, and 21.1% with steroids (p < 0.001 between groups).

Table 4 shows the achievement of final improvement among the studied participants according to the received treatment protocol. The highest number of patients who improved (reduction in seizure frequency) was observed in the KD group (28 patients), followed by the rituximab group (24 patients); however, there was no statistically significant difference among the three treatment lines regarding the number of improved patients.

**Table 4:** Achieving final improvement among the studied participants according to the received treatment protocol Qualitative data are presented as a number (%). Statistical test: Chi-square test. Statistical significance was set at p < 0.05.

Treatment group	No improvement		Improvement		p-value
Received protocol	n	%	n	%	0.277
Ketogenic diet	7	20	28	80	
Rituximab	11	31.4	24	68.5	
Steroid	13	37.1	22	62.9	

Table 5 shows that patients with a positive family history of epilepsy, consanguinity, or multiple seizure types had significantly greater improvement with the KD than other patients (p = 0.012, p = 0.044, and p < 0.001, respectively). On the other hand, other factors did not significantly affect the efficacy of the added KD.

**Table 5:** Achieving final improvement according to demographic characteristics among patients who received the ketogenic diet Statistical test: Fisher’s exact test. Statistical significance was set at p < 0.05.

Variable	No improvement (n=7)	Improvement (n=28)	p-value
Age (years)	5 (1.5-7)	6 (1-11)	0.406
Age at start of treatment	1 year (6 months to 5 years)	10 months (birth to 14 years)	0.505
Sex: Male | Female	6 (85.7%) | 1 (14.3%)	19 (67.9%) | 9 (32.1%)	0.644
Family history: No | Yes	5 (71.4%) | 2 (28.6%)	5 (17.9%) | 23 (82.1%)	12
Consanguinity: No | Yes	3 (42.9%) | 4 (57.1%)	2 (7.1%) | 26 (92.9%)	0.044
Status epilepticus: No | Yes	4 (57.1%) | 3 (42.9%)	5 (17.9%) | 23 (82.1%)	0.055
Multiple convulsions: No | Yes	5 (71.4%) | 2 (28.6%)	1 (3.6%) | 27 (96.4%)	<0.001
Fever: No | Yes	4 (57.1%) | 3 (42.9%)	7 (25%) | 21 (75%)	0.171
Fits during sleep: No | Yes	7 (100%) | 0	28 (100%) | 0	-
Neonatal history normal preterm fits	7 (100%) | 0	27 (96.4%) | 1 (3.6%)	1
Antiepileptic medications			0.652

## Discussion

The study found that prior to enrollment, patients across all treatment groups (KD, rituximab, and steroids) experienced high seizure frequencies, with a median of 7-8 seizures per day. This elevated rate strongly correlated with refractory epilepsy and was much higher than the accepted benchmark of >1 seizure episode per month, as proposed by Seker et al. [10] for defining refractoriness. After treatment, a significant reduction in seizure frequency was observed in all groups (p < 0.001). The rituximab group showed the largest decrease, with a median of 4 seizures per day, compared to 6 per day in both the KD and steroid groups (p < 0.001). In terms of percentage change from baseline, seizure frequency decreased by 42.9% with rituximab, 30.0% with the KD, and 21.1% with steroids (p < 0.001 between groups). These findings align with the study by Abboud et al. [11]. Patients with autoimmune encephalitis responded well to rituximab, irrespective of autoantibody status or initial treatment response. Approximately 60% of patients who did not initially respond to immunotherapy showed favorable outcomes after rituximab treatment. This 42% improvement rate with rituximab is consistent with the findings of Lee et al. [9]. The study highlights the lack of consensus on the duration of immunotherapy, which generally depends on the patient's response. Some patients may require short-term therapy with monitoring, while others may need more aggressive strategies [12].

The mean age of onset for refractory epilepsy in the study was six years, primarily caused by congenital brain anomalies. This aligns with the findings of Rafique et al. [13], who reported a mean age of onset of 5.3 years. Younger age at onset was associated with an increased likelihood of refractoriness, which may be attributed to the high number of gap junctions present in the young brain, resulting in abnormal connectivity and heightened epileptogenicity [10].

The study found a higher prevalence of refractory epilepsy in males (65%), which is consistent with the literature. Males may receive preferential healthcare-seeking in some cultures [14], and epilepsy is generally more common in males due to their increased risk of exposure to lesional and acute symptomatic causes [15]. Additionally, males may experience more secondary seizure spread, SE, and complications like sudden unexpected death in epilepsy (SUDEP) than females [16]. A positive family history of epilepsy was observed in 42% of patients, consistent with the findings of Gupta and Apleton [16], who proposed that a family history of epilepsy in first-degree relatives is positively associated with drug-resistant epilepsy (DRE). The inherited nature of genetic and metabolic disorders that present with epilepsy explains this phenomenon [17]. Consanguinity was positive in 76% of patients, aligning with the studies by Rafique et al. (2021) [13]. Consanguineous marriages are common in certain regions, such as North Africa, the Middle East, and South Asia, and among migrant communities, and are associated with an increased risk of genetic disorders in offspring. SE and multiple seizure types were prevalent in 74% and 80% of cases, respectively, and were identified as significant risk factors for refractoriness. This data is consistent with the findings of Yuan et al. (2018), who reported that SE and multiple seizure types are strong risk factors for DRE [17]. SE can result from decreased inhibition, hyperexcitability, and reduced GABAergic function, contributing to neuronal death. SE duration ≥ 24 hours was an independent predictor of DRE after convulsive SE. Berg et al. (2010) explained that febrile seizures can lead to mesial temporal lobe epilepsy (MTLE), and prolonged febrile seizures during infancy are associated with severe damage to the temporomesial structures, including hippocampal neuron loss and lesions. MTLE with hippocampal sclerosis (MTLEHS) is the most common DRE [18]. In contrast to the study by Beghi (2019), which found focal epilepsy to be the most common type, this study observed a higher frequency of generalized tonic-clonic seizures compared to focal types. This discrepancy may be due to underdiagnosis of other types in low- and middle-income countries, reflecting a lack of recognition and diagnostic tools, as generalized epilepsy is more severe [19,20]. Children with cognitive impairment or long-standing refractory epilepsy may have difficulty describing or recalling focal seizures, leading to the perception of a higher prevalence of generalized seizures [21].

Factors such as positive family history of epilepsy, positive consanguinity, and multiple seizure types showed significant improvement with the KD compared to other treatment groups (p = 0.012, 0.044, and <0.001, respectively). Other factors did not significantly affect the efficacy of the KD. The KD is particularly beneficial for patients with positive consanguinity and multiple seizure types [22], such as those with syndromic epilepsy like Dravet syndrome and tuberous sclerosis [23]. Multiple trials have demonstrated the efficacy of KD in children with Dravet syndrome, suggesting early consideration of this treatment modality [24]. Preliminary evidence from uncontrolled studies in drug-refractory epileptic children has shown total seizure resolution in 16%, greater than 90% seizure reduction in 32%, and greater than 50% seizure reduction in 56% with KD [25]. In a randomized controlled trial by Neal et al. (2008), children receiving early KD had a 38% decrease in seizures compared to a 37% increase in the control group (p < 0.0001) [24,25]. A 2018 Cochrane Review summarized data from 11 randomized controlled trials (RCTs) involving 778 patients (712 children and 66 adults). The maximum seizure resolution and reduction rates observed with the classical 4:1 KD were 55% and 85%, respectively, at three months after therapy initiation.

## Conclusions

The KD showed the greatest overall improvement in the patient population, while rituximab resulted in the most significant reduction in the number of seizures per day. Patients with positive consanguinity, multiple types of convulsion, and a positive family history of epilepsy showed significant improvement with ketogenic drugs compared to other patients.
